# A multivariable analysis to predict variations in hospital mortality using systems-based factors of healthcare delivery to inform improvements to healthcare design within the English NHS

**DOI:** 10.1371/journal.pone.0303932

**Published:** 2024-07-05

**Authors:** Andrew J. Gardner, Søren Rud Kristensen

**Affiliations:** 1 Centre for Health Policy, Imperial College London, London, United Kingdom; 2 William Harvey Research Institute, Critical Care and Perioperative Medicine Research Group, Queen Mary University of London, London, United Kingdom; King Saud University Medical City, SAUDI ARABIA

## Abstract

Over the last decade, the strain on the English National Health Service (NHS) has increased. This has been especially felt by acute hospital trusts where the volume of admissions has steadily increased. Patient outcomes, including inpatient mortality, vary between trusts. The extent to which these differences are explained by systems-based factors, and whether they are avoidable, is unclear. Few studies have investigated these relationships. A systems-based methodology recognises the complexity of influences on healthcare outcomes. Rather than clinical interventions alone, the resources supporting a patient’s treatment journey have near-equal importance. This paper first identifies suitable metrics of resource and demand within healthcare delivery from routinely collected, publicly available, hospital-level data. Then it proceeds to use univariate and multivariable linear regression to associate such systems-based factors with standardised mortality. Three sequential cross-sectional analyses were performed, spanning the last decade. The results of the univariate regression analyses show clear relationships between five out of the six selected predictor variables and standardised mortality. When these five predicators are included within a multivariable regression analysis, they reliably explain approximately 36% of the variation in standardised mortality between hospital trusts. Three factors are consistently statistically significant: the number of doctors per hospital bed, bed occupancy, and the percentage of patients who are placed in a bed within four hours after a decision to admit them. Of these, the number of doctors per bed had the strongest effect. Linear regression assumption testing and a robustness analysis indicate the observations have internal validity. However, our empirical strategy cannot determine causality and our findings should not be interpreted as established causal relationships. This study provides hypothesis-generating evidence of significant relationships between systems-based factors of healthcare delivery and standardised mortality. These have relevance to clinicians and policymakers alike. While identifying causal relationships between the predictors is left to the future, it establishes an important paradigm for further research.

## 1. Introduction

There are clear variations in hospital inpatient mortality across England, which have been reported in the literature [1,2] and national media [3,4]. Determinants of such variations have been previously cited as the number of emergency admissions, the availability of hospital beds, the number of doctors and critical care bed capacity [1,5–8]. A model which could predict a hospital at risk of excess intra-hospital mortality is desirable, in order to deploy finite healthcare resources more effectively. Further, such factors may highlight those NHS Trusts in need of systems-based improvements to their service delivery. Hence, this paper sought to establish associations between intra-hospital variations in the Summary Hospital-level Mortality Indicator (SHMI) and publicly reported variables of hospital activity and resource, and if these relationships change over time. The identified associations highlight the critical relevance of systems factors in determining healthcare outcomes.

### 1.1 Background

Over a century of innovation has revolutionised the scientific understanding of disease and technologies with which to treat it. Independently, the way in which healthcare is delivered to global populations has also been iterated [9]. The growing centralisation of healthcare is an example of this [10]. An idealised health system would ensure available resources are delivered to achieve the best possible population health in the optimal cost-effective manner [11]. However, many barriers prevent this ambition from being achieved, not least by how healthcare systems are designed. A large body of literature has demonstrated the benefits of systems-based approaches internationally [12,13]. These range from improvements in patient outcomes, including reductions in mortality and length of stay, as well as quality of care metrics (e.g. reduced readmissions) [14–19]. However, the significant heterogeneity in study methodology makes the interpretation of these data challenging. These difficulties are compounded by a lack of transparency, which makes interventions hard to reproduce [20,21]. Further, many are either limited to a small region, single site, or even an individual workstream within a hospital (e.g. emergency care) [11,13].

### 1.2 Resource availability may influence mortality and national trends may exacerbate these associations

NHS statistics demonstrate an increasing demand for its services over the last decade, both for emergency and elective care. There is a concern that has and will negatively impact on health outcomes, including mortality. Measures of resource strain are correlated with poorer outcomes [22]. However, determining the contribution of any one factor is challenging, given the complex and diverse factors which affect population-based health outcomes. Addressing this question may be achieved by correlating how metrics of stain and outcomes vary between hospital trusts.

#### 1.2.1 Accident & emergency care

From 2011 to 2019, the volume of patients attending A&E across English NHS trusts increased by almost 30%. The largest factor was a significant rise in non-urgent attendances (rising 114%). Overwhelmed resources result in long waits and overcrowding within departments. Both of these phenomena have been associated with an increase in patient mortality, length of stay, and the delayed treatment of life-threatening medical conditions including pneumonia and myocardial infarction [23–32]. Addressing this, the implementation of waiting time targets in Australia and New Zealand reduced overcrowding, and increased the efficiency of care delivery [33–35]. England’s own four-hour target (FHT) was introduced in 2004 to address historical failings in A&E outcomes. However, its potential benefits have been poorly studied.

The strain on A&E departments from rising attendances is exemplified by a persistently deteriorating FHT performance [36]. The 95% target for assessing and treating patients has not been met in major A&Es since Q1 2012. From 2011 to 2019, the time taken to see 95% of all patients has increased by over a third. This suggests that the maximum wait of some A&E patients has risen substantially. With a similar trend, the number of patients admitted to hospital has increased by nearly a quarter. However, the number of acute hospital beds for them to occupy has decreased Thus, the number of admissions per bed has been exaggerated.

#### 1.2.2 Bed occupancy and length of stay

A greater hospital bed occupancy is linearly associated with a higher rate of adverse patient events (e.g. hospital-acquired infections) [37–40] and mortality [23,41–44]. These effects appear particularly pronounced above an occupancy of 90%. Whilst the quality of the current literature on this topic is limited (for example, no prospective controlled studies have been conducted), the association between the highest bed occupancies and adverse events has been reproducibly demonstrated. The U.K. National Audit Office has thus recommended hospitals should aim for a target of under 85% [45]. Despite this, the average bed occupancy has increased by 2.6%, to 90.6% in 2019.

Length of stay (LOS) has an uncertain relationship with mortality [46–49]. If one exists, it is likely to be weak and plagued by endogeneity. There are conceivable reasons why both an inappropriately short LOS, and an inappropriately long LOS could negatively impact outcomes. For example, respectively due to inadequate treatment and a subsequent need for readmission, or hospital-acquired infection [47].

#### 1.2.3 Intensive care

Intensive Care Units (ICU) provide supportive care for those most critically unwell, though with exceptionally high resource requirements has a limited capacity. Approximately 2% of acute hospital admissions require intensive care [50]. Unsurprisingly, these patients have the highest mortality risk. Like general bed capacity, there is a significant national variation in Trusts’ ICU capacity. This is not necessarily correlated to the number of patient admissions. Value-judgements must be made by intensivists regarding who may benefit from this finite resource [51–53]. A patient is more likely to be admitted to an ICU with greater bed availability [53]. Further, a recent U.K. study demonstrated a decreased ICU bed capacity corresponded with a significantly increased hospital mortality, an association which was most evident for the sickest patients [7,8,54]. Over the last decade, while the average ICU bed occupancy has slightly decreased, there has been an increase in the number of ICU beds. Together, this represents an 8% increase in the number of patients receiving ICU based care.

#### 1.2.4 Staffing

Greater staffing by both doctors and nurses has been associated with better outcomes for patients, including reduced hospital mortality and length of stay [1,55–59]. Along with a general association, this has been particularly demonstrated within the ICU context [7,54,60–62]. While NHS staffing numbers have increased over the last decade, the U.K. remains well below the Organisation for Economic Co-operation and Development (OECD) average for both the number of doctors and nurses per head of population [63].

### 1.3 Stain may not be felt equally

The literature suggests that the strain on healthcare services has increased over the last decade. However, the capacity for different Trusts to address this challenge will vary. These include disparities in the resources available, including both financial and physical assets (for example, ICU beds, radiology scanners and laboratory capacity), along with their workforce [64]. The COVID-19 pandemic has brought to the forefront such concerns, with fewer doctors, nurses, general medical beds, and ICU capacity, all being associated with increased mortality [44,65,66]. Prior to this, implementation of a ‘7-day services’ policy, which aimed to increase hospital resources at the weekend, has been associated with improvements in care quality process metrics, and fewer clinical errors [67]. Those with the least capability, may have borne the excess strain within the health system asymmetrically. This may have had a negative impact on patient outcomes [68].

With this context, it is hypothesised that if associations exist between systems-based resource factors and the variation in mortality between Trusts, these will have strengthened over time. In order to address this, and hence the research question of this study, potential predictors of the variation in mortality between Trusts needed to be identified.

### 1.4 Predictors of mortality variance

Highlighting the interrelatedness of system-factors which may influence patient outcomes, studies have demonstrated how poor patient flow at discharge results in a higher bed occupancy–and these are associated with a worse FHT performance and higher inpatient mortality [42,69,70]. Further, such associations are dynamic: if flow measures improve, these associations diminish [71]. This demonstrates the challenge in finding predictors which are independent of one another, and also the importance of a multivariable approach in linear regression.

A substantial quantity of high-quality hospital and Trust-level activity data is now published by the NHS, including the Summary Hospital-level Mortality Indicator (SHMI), a standardised metric of hospital mortality [72]. Potential predictors of SHMI selected for inclusion into univariate and multivariable linear regression analyses. These were identified with consideration of the wider literature, along with the aforementioned resource factors [1,49,71,73].

Although this approach will be unable to establish causation, any correlations between the predictors and SHMI can be important indicators for further examination. These will be useful for both trusts and policymakers alike. For the former, whether they are outliers in terms of a given systems-based factor, as this may influence their outcomes. For the latter, helping to inform standard setting for systems-based resources.

After further investigation to establish causality, such predictors could be incorporated into a predictive risk model of mortality. Models are useful for policymakers and clinicians alike, as a means of synthesising, quantifying and then benchmarking healthcare performance [74]. Current statistical surveillance tools have flaws, including a lack of transparency and an arbitrary design [75–79]. Particularly, the Care Quality Commission (CQC) tool, ‘Intelligent Monitoring’, has been shown poorly predictive of both qualitative and quantitative outcomes [80,81]. This study may serve to benefit an iteration in the current provision.

## 2. Methods

### 2.1 Data collation

Data from English NHS trusts were accessed from open datasets hosted on the NHS Statistics and NHS Digital websites [72,82]. Complete data were available from quarter 1, 2011. The onset of the coronavirus pandemic and its subsequent impact on healthcare data confounded the use of data after quarter 2, 2020. Thus, the analysis was limited to the full years 2011 to 2019. The following statistical reports were collated: “Emergency Department attendances & emergency admissions”; “Hospital Episode Statistics”; “NHS workforce statistics”; “Hospital activity statistics”; “Critical Care bed capacity”; “Bed availability and occupancy”; “Summary Hospital-Level Mortality Indicator (SHMI)”. Full definitions and submission guidelines for each indicator are available from the aforementioned sources. Each NHS trust takes responsibility for the routine reporting of its activity, which is then published centrally.

### 2.2 Data filtering

Collated data were filtered in order to only capture acute emergency adult hospital admissions, which represent the vast majority of in-hospital deaths. Thus, trusts were excluded if they did not accept urgent (type 1) A&E patients or have critical care facilities. Likewise, non-NHS providers, NHS specialist trusts (including hospital admissions to maternity, paediatric, mental health and learning disability beds), walk-in or treatment centres and community providers were all excluded. To avoid comparing significantly different trusts in terms of activity, trusts were excluded if they admitted under 1000 patients per year. Finally, trusts reporting less than 50% of variables within a statistic report were excluded for that year. These were often at the dissolution or formation of new trusts over the decade.

### 2.3 Variables

The included variables were either absolute values or a ratios derived from data covering the same time-period, at the same Trust (e.g. ICU beds per admission). For standardisation, all variables were created by aggregating quarterly data into yearly averages, unless they were already in this format. A trusts SHMI was used as the outcome (dependent) variable with which to assess the variation in intra-hospital mortality. Predictor (independent) variables were compared against the variance in SHMI, having been identified from the collated statistical reports. The rationale for those selected described is described within S1 Appendix 1 in S1 File. Of note, previous studies have shown LOS to be non-normally distributed [46,48], yet the available data only permitted the use of the mean LOS. Despite its distribution, some authors advocate for the use of the mean irrespective as it would account for the resource burden prolonged admissions represent [83].

SHMI is a standardised measure of hospital mortality. It is the ratio of the actual deaths following a hospital admission and the expected deaths given the clinical casemix and characteristics of the patient population [82]. A score is assigned between 0 and 2, with the English average standardised to a score of 1.000 [84]. A SHMI under 1 indicates fewer deaths were observed than expected, while to opposite true for an SHMI over 1. Given that demographics are included within SHMI, these were not considered as potential predictors. Despite potential flaws, it is the only nationally available government statistic of hospital mortality variance [1,85–87].

### 2.4 Timepoints

The data from three equally space years across the 2011–2019 reference period were selected on which to perform cross-sectional regression analyses. This approach was taken to provide a representative sample, helping to limit the effect of any confounding regional or national phenomena. It equally reduces the statistical concerns of multiple testing and is consistent with the resources available to conduct the study.

### 2.5 Bias

NHS reorganisation over the decade, largely through mergers, resulted in a diminishing number of trusts included within each analysis. In order to account for this potential bias, the analysis was repeated to only including those trusts common to all three analysis years. This meant for 2019, 2015 and 2011, that 4.9%, 12.0% and 15.2% of trusts were excluded from the Robustness test, respectively. Across each analysis year SHMI was the most frequently missing datapoint, but for no more than for 5% of trusts in each analysis year. All other variables were missing for no more than 2.5% of trusts.

### 2.6 Statistics

Descriptive statistics were used to report the change in absolute values overtime. Histograms’ bin sizes were determined to provide an approximately equal distribution (appendices 2 & 4). These were then kept consistent across all years of comparison.

For the univariate analysis (UVA), ordinary least squares linear regression was conducted using SHMI as the dependent (outcome) variable, against each of the independent (predictor) variables. A series of cross-sectional regression analyses were performed for each year separately. Results are presented for each analysis with the statistical significance value (judged as p = <0.05), regression coefficients and the coefficient of determination (R-squared (R^2^)). The statistical principles of inclusivity and simplicity were balanced. Thus, statistical significance within the UVA did not automatically exclude the predictor from the multivariable analysis (MVA). However, if the variables continued to add little strength to the model they would be removed, and a reanalysis undertaken.

Assumption testing and robustness analyses were conducted in order to ensure the internal validity of the MVA (S1 Appendix 2 in S1 File). The data were assessed for multicollinearity, independence of the residuals, linearity, homoscedasticity and normality. Anomalies are reported in the results section. Outlying trusts, identified using Cook’s distance, central leverage values and Mahalonobis distance, were excluded and the MVA repeated.

The analysis conducted for this paper was completed using Microsoft Excel (v.17, 2019), IBM SPSS Statistics (v.26) and Stata (v.17).

## 3. Results

Over the 2011 to 2019 analysis period, the number of acute hospital trusts with A&E departments fell from 149 to 133. The majority of these were mergers of a number of small Trusts into a much larger one. Missing data further reduced the included trusts, such that only 138, 133 and 123 trusts for 2011, 2015 and 2019, respectively, were included in the regression analysis (Table 1). The number of trusts common to all three years, and those on which the robustness analysis was completed, was 117.

**Table 1:** Descriptive statistics of the independent and dependent variable(s), correct to 3 significant figures.

		2011	2015	2019
n		138	133	123
**SHMI**	Mean (Std. Dev)	1.00 (0.098)	1.00 (0.090)	1.00 (0.101)
	Median (Q1-Q3)	1.02 (0.944–1.06)	1.00 (0.960–1.07)	1.01 (0.958–1.07)
	Min-Max	0.71–1.25	0.68–1.18	0.69–1.20
**Bed Occupancy**	Mean (Std. Dev)	87.5 (5.29)	89.3 (5.00)	90.2 (4.27)
	Median (Q1-Q3)	87.9 (84.6–91.4)	89.6 (85.9–92.9)	90.7 (87.6–93.0)
	Min-Max	62.7–96.9	69.2–99.0	77.9–98.0
**FHATB**	Mean (Std. Dev)	96.7 (4.28)	90.5 (8.22)	81.7 (12.1)
	Median (Q1-Q3)	98.0 (95.6–99.6)	92.5 (85.8–96.6)	83.7 (73.8–90.6)
	Min-Max	68.1–100	55.3–100	41.8–100 (58.2)
**ICU beds per 10,000 admissions**	Mean (Std. Dev)	6.96 (5.36)	6.51 (4.55)	5.69 (4.22)
	Median (Q1-Q3)	5.33 (3.89–7.98)	4.72 (3.60–8.20)	4.01 (3.33–6.08)
	Min-Max	2.20–35.5	2.77–31.7	2.15–28.57
**Doctors per bed**	Mean (Std. Dev)	0.827 (0.223)	0.896 (0.235)	1.03 (0.281)
	Median (Q1-Q3)	0.771 (0.691–0.899)	0.830 (0.740–0.995)	0.95 (0.830–1.16)
	Min-Max	0.445–1.91	0.560–1.71	0.590–2.05
**Nurses per bed**	Mean (Std. Dev)	1.76 (0.434)	2.01 (0.511)	2.17 (0.544)
	Median (Q1-Q3)	1.63 (1.48–1.91)	1.87 (1.66–2.28)	2.01 (1.75–2.42)
	Min-Max	1.03–3.58 (2.55)	1.32–4.68 (3.36)	1.48–4.34 (2.86)
**Mean LOS**	Mean (Std. Dev)	4.23 (0.583)	4.20 (0.600)	4.02 (0.605)
	Median (Q1-Q3)	4.20 (3.80–4.50)	4.17 (3.75–4.49)	3.97 (3.65–4.29)
	Min-Max	3.00–6.20	2.85–6.11	2.67–6.07

Bed occupancy and the four-hour admit to bed are presented as percentages. Mean length of stay (LOS) in days. Doctors and nurses (headcount of staff members of that group) per bed. SHMI as the derived standardised figure as previously described. SHMI: Standardised Hospital Mortality Indicaotr, FHATB: Four Hour admit to bed, ICU: Intensive Care Unit.

### 3.1 Descriptive analysis

Table 1 quantifies the change in each variable between 2011 and 2019, mirroring the national trends described previously. While bed occupancy and the number of doctors and nurses increased, the FHATB performance and ICU beds per 10,000 admissions (‘ICU beds’) decreased. Notably, there was a marked increase in the interquartile range of the FHATB (four-fold).

Each variable was assessed visually (S1 Appendix 4 in S1 File). As the dependent variable (SHMI) is not significantly skewed or kurtotic there is no indication to perform non-linear regression.

### 3.2 Univariate analyses

Table 2 shows the result of the ULR analyses of each predictor using SHMI as the dependent variable, for the years 2019, 2015 and 2011.

**Table 2:** The results of the univariate analyses comparing the independent (predictor) variables to the dependent variable (SHMI: Standardised Hospital Mortality Indicator).ICU: Intensive Care Unit, Unstd.

		2011	2015	2019
**Four-hour admit to bed**	Unstd. Reg. Coeff.	-0.000489	-0.00191	-0.002
	(95% CIs)	(-0.00437 to 0.00339)	(-0.004 to -0.00005)	(-0.004 to -0.001)
	Significance (p-value)	0.804	0.044	0.002
	R^2^	0.000	3.0%	7.3%
**Bed occupancy**	Unstd. Reg. Coeff.	0.003	0.003	0.006
	(95% CIs)	(0.000 to 0.006)	(0.000 to 0.006)	(0.002 to 0.010)
	Significance (p-value)	0.037	0.032	0.004
	R^2^	3.2%	3.4%	6.8%
**ICU beds per 10,000 adm.**	Unstd. Reg. Coeff.	-0.007	-0.006	-0.010
	(95% CIs)	(-0.010 to -0.004)	(-0.010 to -0.003)	(-0.014 to -0.006)
	Significance (p-value)	<0.001	<0.001	<0.001
	R^2^	14.1%	10.4%	16.5%
**Doctors per bed**	Unstd. Reg. Coeff.	-0.257	-0.213	-0.204
	(95% CIs)	(-0.317 to -0.196)	(-0.268 to -0.158)	(-0.257 to -0.150)
	Significance (p-value)	<0.001	<0.001	<0.001
	R^2^	34.2%	30.6%	32.1%
**Nurses per bed**	Unstd. Reg. Coeff.	-0.093	-0.046	-0.062
	(95% CIs)	(-0.128 to -0.058)	(-0.076 to -0.017)	(-0.093 to -0.030)
	Significance (p-value)	<0.001	0.002	<0.001
	R^2^	17.0%	6.9%	11.0%
**Mean lenght of stay**	Unstd. Reg. Coeff.	0.026	0.016	0.027
	(95% CIs)	(-0.002 to 0.054)	(-0.010 to 0.042)	(-0.002 to 0.057)
	Significance (p-value)	0.071	0.229	0.070
	R^2^	2.4%	1.1%	2.7%

Reg. Coeff.: Unstandardised regression coefficient, CI: Confidence interval.

For 2019 and 2015, all apart from the mean LOS were statistically significant. In 2011, SHMI’s association with bed occupancy and the FHATB lacked significance. This may reflect a rise in bed occupancy and deterioration in FHATB performance observed over time (Table 1).

The greatest R^2^ magnitude was with the number of doctors per bed. Each variables’ association with SHMI is largely consistent over time, although notably the FHATB and bed occupancy display an increasing trend. Over 2011 to 2019, this was four-fold and three-fold, respectively, albeit of a comparatively low magnitude throughout. Nurses per bed appeared to have an anomalously low association in 2015.

The UVA predicts that an additional 1 doctor per bed would be associated with a fall in SHMI of 0.257. In terms of their trends, the unstandardised coefficient size of doctors, nurses and the mean LOS remain stable over time. In contrast, the size of the ICU beds and FHATB coefficients increase over time, and therefore these predictors are associated with an increasingly negative effect on the SHMI (Table 2).

**Fig 1:**
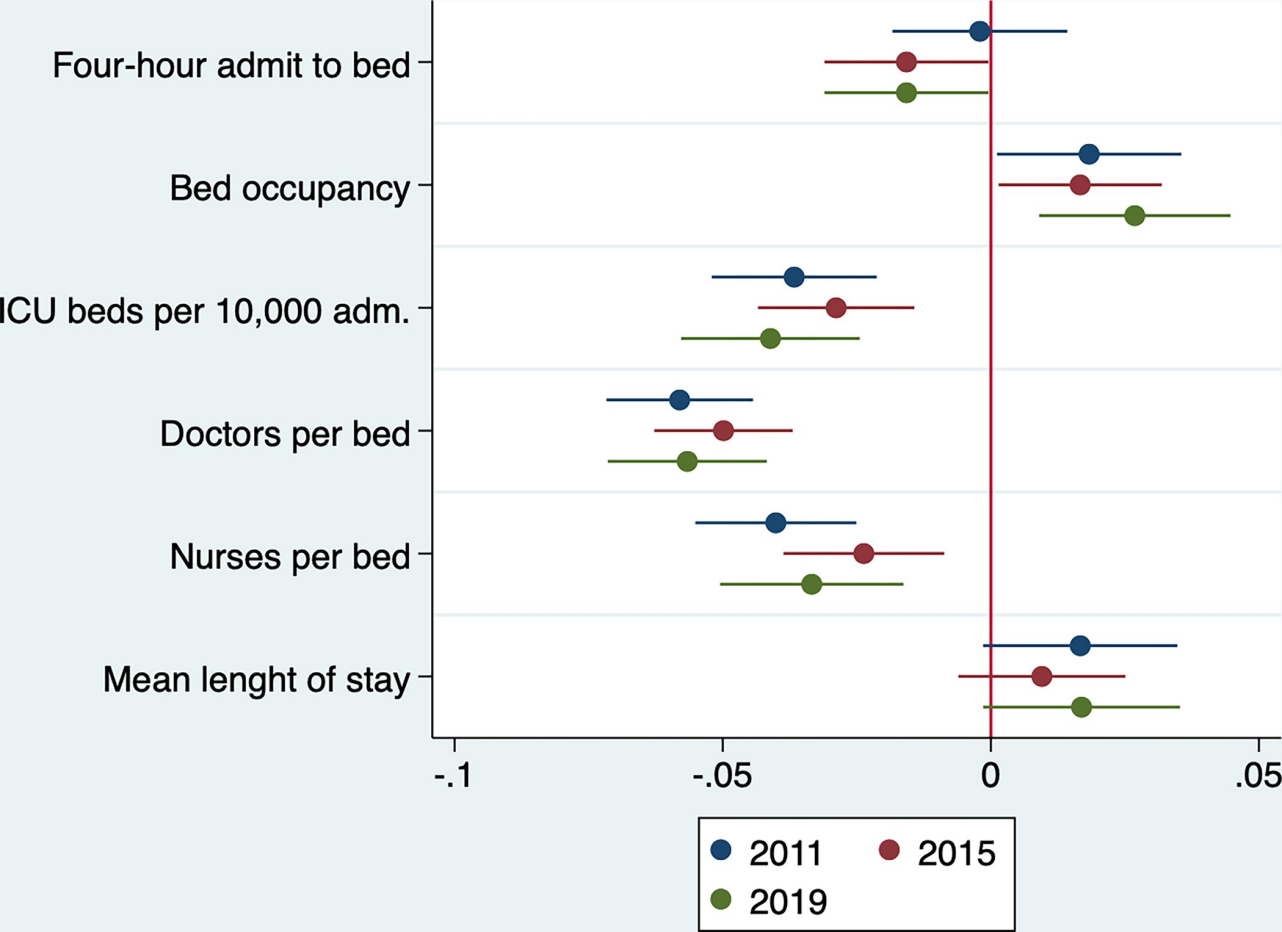
Comparison of standardised regression coefficients from univariate models.

The standardised coefficients displayed in Fig 1 follow a similar trend with the largest effect on SHMI by doctors per bed, followed by ICU beds, and then nurses per bed. Mean LOS has the least effect. This pattern is identical for 2015, and similar for 2011. For 2011, the exceptions compared to the other years are that nurses per bed have a greater effect size than ICU beds, and the FHATB has almost no effect at all. The positive or negative influence on SHMI by an individual predictor remain consistent across all three years.

### 3.3 Multivariable analyses

As described, two sequential MVA were undertaken. Firstly, to include all UVA predictors in the model (S1 Appendix 5 in S1 File), and secondly to exclude those which were not consistently statistically significant on UVA (i.e. mean LOS) (Table 3). Thus, 6 or 5 predictors were used in each MVA (termed ‘6P’ and ‘5P’), respectively.

**Table 3:** The results of the 5-independent predictor ‘5P’ multivariable regression analysis, comparing their association with SHMI.

		2011	2015	2019
**Four-hour admit to bed**	Unstd. Reg. Coeff.	0.000462	-0.00134	-0.00151
	(95% CIs)	(-0.00270 to 0.00362)	(-0.00290 to 0.000216)	(-0.00276 to -0.000255)
	Stnd. Reg. Coeff.	0.0202	-0.122	-0.1805
	Significance (p-value)	0.773	0.091	0.019
**Bed occupancy**	Unstd. Reg. Coeff.	0.00379	0.00291	0.00452
	(95% CIs)	(0.00124 to 0.00635)	(0.000354 to 0.00546)	(0.000897 to 0.00814)
	Stnd. Reg. Coeff.	0.205	0.163	0.190
	Significance (p-value)	0.004	0.026	0.015
**ICU beds per 10,000 adm.**	Unstd. Reg. Coeff.	-0.000422	-0.0000886	-0.00222
	(95% CIs)	(-0.00362 to 0.00277)	(-0.00352 to 0.00335)	(-0.00667 to 0.00222)
	Stnd. Reg. Coeff.	-0.0230	-0.00445	-0.0926
	Significance (p-value)	0.794	0.959	0.324
**Doctors per bed**	Unstd. Reg. Coeff.	-0.304	-0.227	-0.231
	(95% CIs)	(-0.409 to -0.200)	(-0.301 to -0.153)	(-0.317 to -0.146)
	Stnd. Reg. Coeff.	-0.693	-0.590	-0.644
	Significance (p-value)	<0.001	<0.001	<0.001
**Nurses per bed**	Unstd. Reg. Coeff.	0.0335	0.0157	0.0453
	(95% CIs)	(-0.0164 to 0.0834)	(-0.0145 to 0.0459)	(0.00676 to 0.0838)
	Stnd. Reg. Coeff.	0.148	0.0887	0.243
	Significance (p-value)	0.187	0.306	0.022
**R** ^**2**^		38.9%	35.6%	42%
**Model sig. (p-value)**		<0.001	<0.001	<0.001

Unstd. Reg. Coeff.: Unstandardised regression coefficients. Stnd. Reg. Coeff: Standardised regression coeffciient Sig.: Significance.

The number of doctors and bed occupancy demonstrated statistical significance in each year, with negative and positive associations with SHMI, respectively. The FHATB and nurses were only significant in 2019. ICU beds were not significant in at any timepoint, nor the mean LOS within the ‘6P’ analysis. When comparing the standardised coefficients, doctors per bed clearly represent the strongest association with SHMI variation. This is broadly consistent across all three years.

The coefficients for bed occupancy and nurses per bed are approximately stable, while ICU beds and the FHATB become increasingly negative. Comparing these two, while the change is four-fold for ICU beds, it is nearly 10-fold for the FHATB. This marked change is represented equally in the UVA and descriptive analysis. The coefficients for bed occupancy are similar within the UVA in terms of sign and size, while those for ICU beds are much reduced. However, nurses per bed change in both these regards in the MVA. While having a consistently negative sign in the UVA, it is positive within the MVA and the coefficient size increases over time (MVA average 0.0315 vs. UVA -0.0663) although the variable is only statistically significant in 2019.

The variation in SHMI explained by the chosen predictors is approximately 36%. The R^2^ value of each model is broadly consistent both between years and methodologies (‘6P’ vs. ‘5P’), with a slightly increasing trend over time. The strengthening associations in the FHAB and ICU beds may reflect the concurrent rise in the R^2^. The removal of the mean LOS from the MVA has little effect on the R^2^ value. However, a marginal increase in the magnitude of nearly all the predictors’ coefficients is observed.

### 3.4 Robustness testing

In order to ensure demographic variables were adequately controlled for within the SHMI algorithm, these variables (including age and gender) were individually analysed by univariate regression, and then sequentially included within the 6P MVA. In no circumstance were the variables statistically significant in regression, nor did they change the adjusted R-squared significantly (results not shown).

To discount the effect of outliers (identified using the methods described above), the MVA was repeated with these trusts excluded (S1 Appendix 6 in S1 File). For each year, the R^2^ is somewhat reduced but those predictors which were statistically significant remained the same, and equally the standardised coefficients are similar. Therefore, it appears that the presence of these concerning outlying entries did not have an egregious effect on the results of the MVA.

Secondly, it is possible that the changing composition of hospital trusts over time could have affected the observations of the analysis. To exclude this, the MVA was repeated on only the 117 common to all three years (S1 Appendix 7 in S1 File). Again, these results are broadly consistent with our original analyses, although for 2015 the FHATB and bed occupancy gain and loose statistical significance, respectively. The R^2^ increases in the common set MVA for each year, and particularly so for 2011. This could be accounted for by an increase in the effect size of doctors per bed. While small, this predictor has the greatest size and thus the marginal effect may be exaggerated compared to others.

These data would suggest that the associations observe within the MVA were not affected by the changing composition of trusts, nor those which are statistical outliers. Indeed, some element of the associations may have been masked.

## 4. Discussion

### 4.1 Summary of findings

Over three sequential cross-sectional retrospective analyses, the 5 predictors within the MVA were able to reproducibly predict approximately 36% of the variation in SHMI between hospital trusts. While this leaves 64% in variance unaccounted for, the model performs well comparative to similar studies within the literature [71]. Speculatively. remaining factors may be found within the macro-healthcare environment, such as efforts to centralise services, or the availability of ambulance and social care services. Equally, this unexplained variance could be due to individual hospital factors, like the skill, morale and leadership of staff, or the availability of unmeasured resources within our model (e.g. radiology, theatre space). The number of doctors per bed was most robustly and consistently associated with SHMI, with the bed occupancy secondary to this. The FHAB metric became more strongly associated with SHMI over time. While ICU beds per 10,000 admissions and nurses per bed were significantly associated with SHMI on UVA, this was not the case when included within the MVA. Inclusion of the mean LOS did not benefit the model, and thus was excluded. Assumption testing of the MVA, and the robustness analyses support the internal validity of these findings. Sole inclusion of the trusts common to all three years strengthens the overall association between the predictors and SHMI. The average R^2^ increased from 36% to 0.39%. It is possible that during the reorganisation of trusts there is an exaggerated oversight and surplus of resources, such that a degree of the association of the predictors and SHMI is masked.

### 4.2 Doctors per bed

Doctors have a critical role in healthcare delivery, from diagnosis to clinical intervention. Hence, that the number of doctors determine the greatest variation in SHMI seems plausible. Jarman *et al*., utilising similar methodology, equally showed that the number of doctors per bed was the best predictor of an excess standardised hospital mortality for emergency admissions. They found that a 1% increase in the number of hospital doctors per bed was associated with a 0.119% decrease in hospital standardised mortality (equating to 333 more doctors and 186 less deaths). The impact on doctor staffing and negative patient outcomes, including mortality, has been linked by other studies within the literature [56,88–90].

The NHS may have an exaggerated vulnerability to a natural variation in doctor numbers between hospitals, given their comparative scarcity compared to other westernised healthcare systems. Aside from Poland, the U.K. has the lowest number of doctors per population in the European. Compared to the E.U. average (3.7), OECD average (3.5), and other western European countries such as Germany (4.25), Spain (4.6) and Norway (5.2), the U.K. lags significantly behind at 2.81 doctors per 1000 inhabitants [63].

The increase in total doctors nationally (Table 1) by around 20%, may have offset other factors which have potentially strained patient care over the decade. For example, patient acuity has also risen over time and heavy clinical workloads are an important determinant of unsafe care [91,92]. Related with this, patient throughput per bed has increased with admissions having risen yet hospital beds and the average lengths of stay have diminished. Broadly, between UVA and MVA, as well as the robustness analyses, the coefficients remain of a similar sign and size. It is possible the increase in doctors over time helped equalise the differences in doctor numbers between trusts, which may account for the slightly higher coefficient in 2011. Finally, the effect of the 2015 junior doctors contract dispute on the slightly lower coefficient in 2015 is uncertain. One possibility is that as consultants adopted a more ‘hands-on’ role, this may have actually represented an excess of doctors, given the number of consultants has risen significantly more than non-consultant (junior) doctors.

Given its average correlation with nurses per bed (~r = 0.692, S1 Appendix 8 in S1 File), the interpretation of this predictor’s coefficients must be approached with caution. Distinguishing their relative effect on the variation of SHMI is challenging and requires further investigation. This limitation will be discussed in more detail further into the discussion.

### 4.3 Bed occupancy

An increasingly high bed occupancy is associated with worse outcomes. This effect is especially pronounced above 90% [23,37–44], which became the national median in 2016. As hospital beds have become fuller nationally, the interquartile range of this metric has narrowed. The metric has come to be more normally distributed and significantly less skewed and kurtotic. This may have benefited the metric remaining to be a good discriminator of mortality performance.

Consistent with the hypothesis that bed occupancy is associated with worse outcomes, a positive predictive relationship is observed between the coefficient and SHMI. Over time its effect size remains of a similar magnitude and is consistently statistically significant. These observations are mirrored within the UVA. However, comparatively its effect size is roughly a third of doctors per bed. Just like doctors, the U.K. has a comparatively low number of hospital beds, having the second lowest compared to E.U. countries. For example, while Germany has 7.9 beds per 1000 population, the U.K. has 2.4 (the OECD average is 4.7) [63].

### 4.4 The four-hour admission to bed (FHATB)

It is well established that patient crowding within A&E negatively effects outcomes for patients requiring admission [23,25–27], and especially so for those critically unwell [93–97]. Thus, it would be expected that a worse FHATB performance would be reflected in excess mortality. Our findings support this hypothesis.

Nationally, the average FHATB performance has significantly worsened over the decade (Table 1). During this period, the metric became increasingly normally distributed and, by 2019, a statistically significant predictor of SHMI with an increasingly negative coefficient (with an effect size matching bed occupancy). Within the common set analysis, the coefficient sizes are larger and the metric is also statistically significant in 2015. One possibility is that any reorganisation of trusts increases the resources and oversight on A&E performance. Thus, newly created trusts mask the true association between FHATB and SHMI.

On UVA the FHATB coefficient is greater than the MVA in 2015 and 2019, suggesting that other predictors were accounting of some of the effect size. Although bed occupancy is on average relatively poorly correlated with the FHATB (r = ~0.211, S1 Appendix 8 in S1 File), having more available beds should ostensibly assist A&E flow. Keogh *et al*. demonstrated a statistically significant relationship between a lower bed occupancy and FHT performance [71]. However, the association between flow variables and the FHT performance was weak (R^2^ ~0.15). While not specifically addressed, given the hypothetical benefits of the FHATB over the FHT, future models should consider its inclusion as an important metric of hospital performance.

### 4.5 ICU beds per 10,000 admissions

Intensive care treats those patients whom are most sick within the hospital. It has previously been shown within the literature that ICU bed availability can impact patient outcomes, including mortality [7,8,54]. Our data supports this, showing that fewer ICU beds adversely affects mortality. As ICU beds have diminished nationally (a 33% median fall nationally, 2011-‘19), the strength of this relationship has increased. However, it remains small compared to the other predictors. ICU beds are strong and statistically significant determinant of SHMI on UVA, however it lacks both these qualities in MVA. Other predictors may thus explain the variation. Second to doctors and nurses, ICU beds are correlated to both these two predictors (S1 Appendix 3 in S1 File). It seems plausible that a well-staffed hospital overall is also reflected in its ICU. It has been demonstrated that better staffing in ICU of physicians and nurses are associated with reduced mortality and LOS, both within ICU and hospital-wide [54,55,60]. Accordingly, the effect of the staffing variables on SHMI may be exaggerating the strength of the association between ICU beds and SHMI on UVA. A further analysis may consider addressing these concerns by removing or substituting this predictor, for example, with ICU bed occupancy, or specific ICU staffing rates. Finally, the extensively skewed and leptokurtic distribution may hinder the ability for this metric to be a discriminator of SHMI performance (S1 Appendix 4 in S1 File). However, if ICU bed resources decreased further, the relationship between ICU beds and SHMI may strengthen.

### 4.6 Nurses per bed

Like doctors, the number of nurses per bed has increased over time (Table 1). However, in 2017, the Royal College of Nurses released a report stating that over half of their survey respondents reported a shortfall in planned staff per shift, and that patient care was compromised because of it [98]. These sentiments are reflected in low overall OECD-reported nursing figures per population, relative to northern European comparator countries [63].

While on UVA, nurses per bed is negatively associated with SHMI and is statistically significant, the opposite is true within the MVA. Counterintuitively, this would suggest that more nurses per bed are associated with excess observed deaths. Despite outliers being excluded, this pattern persists.

This result lacks external validity, given the unlikeness that an excess of nurses are resulting worse patient outcomes. It is likely the predictor is markedly non-independent of one or more of the others, causing multicollinearity. The doctors per bed metric is most likely at fault, as their correlation is the highest between all the predictors (described in S1 Appendix 2 in S1 File, assumption #3). It is higher than the original dataset prior to data filtering. A hospital endowed with doctors is also likely to be with nurses, and this is potentially truer of acute hospital trusts. The pattern of 2011 and 2019 having the highest positive coefficient, matches the two years when the correlation with doctors is also highest. Secondly, the numbers of nurses and ICU beds are also modestly correlated (r = ~0.6), likely given the mandated minimum staffing per patient in ICU [99]. Hence, these predictors may also demonstrate a degree of non-independence.

An additional factor may be that the reported number of nurses are a poor representation of those which are patient facing and are likely to influence patient outcomes. For example, there are a much greater diversity of nursing roles, with many not involved in acute care (e.g. day surgery). Thus, the nursing number’s influence on SHMI may be obscured. While there is a broad literature associating diminished nursing staffing and worse patient outcomes [54,56–61,100–102], few studies rigorously control for the effects of other hospital resource factors (e.g. doctor or bed numbers). Future research is required to disentangle their respective effects on patient outcomes. Finally, a high nurse to patient ratio may indicate a sicker patient population which could further help explain this finding.

### 4.7 Strengths and weaknesses of the study

We believe this study first to examine the relationship between the inter-trust variance in SHMI and systems-level metrics of hospital resource. Furthermore, it elucidates how these relationships have changed (or remained stable) over time. The use of publicly available data helps ensure the relevance and transparency of the analysis. While the explanatory power of the analysis is modest (An MVA R^2^ of ~0.36), it remains stable over time and is commensurate to similar studies. It is plausible the addition of novel variables may improve the model.

The SHMI, the outcome variable used in this study, has been subject to criticism due to its methodology for case mix adjustment [103] and its relevance as a summary performance measure has been questioned [104]. A literature review examining the relationship between various process measures of care and risk-adjusted mortality in general (not the SHMI specifically) found positive associations in 26 out of 51 cases [103]. At a methodological level, except for the three year time frame and case mix adjustment, the SHMI does not explicitly address sampling variability. The SHMI specifically was not found to be associated with avoidable deaths identified on the basis of retrospective case reviews [105], while more recent studies have demonstrated associations between SHMI and components of the British General Medical Council’s junior doctor satisfaction survey [106] and staff-, (but not patient) -satisfaction as expressed in a survey known as the Friends and Family Test [107]. Indeed, the SHMI continues to be published and presented by NHS England as a tool for hospitals, regulators and commissioners to compare and investigate mortality outcomes [108]. Although we fully acknowledge the limitations of the SHMI as a summary indicator of hospital quality, we therefore still feel it has merit as an indicator of one important dimension of hospital quality, and invite other researchers to extend our analysis to other performance measures.

Like all macro analyses of healthcare data, there are inherent limitations in our study. Firstly, relevant data may have been excluded when the datasets were filtered. Trusts with greater resource constraints may be predisposed to submit incomplete data, which may bias the dataset on which the analyses were completed. Equally, poor data quality or ‘gaming’ may both mask and/or exacerbate the relationships identified [71,109]. A limited data quality may limit the reported associations. However, given these data are verified and published by government the study has assumed their veracity. More generally, the 2012 Health and Social Care Act and 2015 junior doctors’ contract dispute may have confounded the relationships observed. It is important to note that the analysis was conducted at trust-level, and thus the associations uncovered are not necessarily found at the individual level.

The analyses was only conducted in three representative years over the analysis period (beginning, middle and end). This was done to limit the multiplicity of comparisons and resource constraints. However, the analysis of additional years of data may have revealed different trends in the data. The temporal scope of the study was limited as robust datasets were not available for all predictors prior to 2011, and limited past 2019 due to the coronavirus pandemic. Floor and ceiling effects of the included variables cannot be discounted; especially regarding bed occupancy and the FHATB where the metrics have approached or reached 100%. Non-linear relationships between mortality variance and these predictors have not been thoroughly investigated, which may lead to threshold effects (i.e. only over/below a certain value is mortality affected in any way). Some predictors may be limited in their discriminatory ability due to the limited distribution of results (e.g. FHATB, ICU beds). As has been previously commented, as with any complex system, ostensibly independent variables may have some degree of overlapping effect. Finally, there is an unclear relation between statistical significance and clinical relevance. An important caveat when interpreting our findings is that our study was not designed to establish causality. Although we have discussed the plausibility of a causal relationship between SHMI and the independent variables, we emphasise that our study is first and foremost exploratory and further research is needed to determine whether causality can be inferred.

The analyses passes assumption testing and robustness checks, suggesting good internal validity. However, the minor drawbacks described, including a degree of non-linearity between some independent and dependent variables (S1 Appendix 2 in S1 File) may have affected the reported relationships. The pattern and size of coefficients largely remaining unchanged. Our data demonstrates the utility of such studies in demonstrating the potential benefit of system-wide policy change. However, there is an important need for prospective and controlled trial designs to investigate the effects of systems-based factors on patient outcomes. These are especially important if attempting to justify the financial cost of policy interventions (e.g. additional hospital beds, trained doctors) in the relatively resource constrained environment of a public healthcare system [100].

### 4.8 Policy implications and further research

Evidence-based policymaking has become established as the gold standard approach. Studies should not only produce the type and strength of evidence that is necessary to justify a change in policy, but also produce practical solutions. This study aimed to directly address these needs. For clinical leaders in healthcare, this research aims to elucidate the systems factors may be negatively impacting patient outcomes. Future research is required to establish whether associations observed represent causal relationships and how they may be included in a predictive model. Contextualising the study within local service demands may provide the fidelity to optimally substantiate service reconfiguration and undertake cost-effectiveness analyses of changes to the service.

## Supporting information

S1 File (DOCX)

## Abbreviations

A&E: Accident & Emergency
FHT: Four-hour target
FHATB: Four-hour admission to bed target
SHMI: Summary Hospital-level Mortality Indicator
ICU: Intensive Care Unit
OECD: Organisation for Economic Co-operation and Development
M/ULR: Multivariable/Univariate linear regression
M/UVA: Multivariable/Univariate analysis
