# Supplementary material for: A multivariable analysis to predict variations in hospital mortality using systems-based factors of healthcare delivery to inform improvements to healthcare design within the English NHS
Source: PLoS One. 2024 Jul 5;19(7):e0303932. doi: 10.1371/journal.pone.0303932 (PMC11226030; doi:10.1371/journal.pone.0303932)
Supplement: S1 File — (DOCX) [file pone.0303932.s001.docx]

Appendix 1: A summary of the rationale for chosen predictors analysed

|  | **Possible Variable** | **Result** | **Explanation** |
| --- | --- | --- | --- |
|  | **Admission load** | | |
| 1 | FHT breached attendances (Type 1) | Excluded | Not all are admitted and therefore may not faithfully impact SHMI |
| 2 | FHT Breached Attendances (All) | Excluded | Similarity to (1) |
| 3 | Four-hour admission to bed (FHATB) | Included | The sickest patients, vulnerable to the negative effects of A&E strain |
| 4 | Conversion Ratio | Excluded | Does not effectively account for volume and lacks clinical relevance |
| 5 | Admission casemix ratio | Excluded | Elective admissions are dealt with in a different pathway |
|  | **Overall hospital bed capacity** | | |
| 6 | Overnight bed occupancy | Included | Previous studies associate this with worse hospital outcomes |
| 7 | Emergency bed load ratio | Excluded | Non-independent of bed occupancy |
| 8 | Total Beds | Excluded | Non-independent of hospital size |
| 9 | Mean length of Stay | Included | An uncertain, yet plausible, effect on patient flow and mortality |
| 10 | Median Length of Stay | Excluded | Data limitations |
|  | **Intensive Care Unit (ICU) Capacity** | | |
| 11 | ICU bed number | Excluded | Non-independent of hospital size |
| 12 | ICU bed occupancy | Excluded | Lacks clinical relevance |
| 13 | ICU capacity per emergency admission | Included | Relevance to literature, relative per volume of admissions |
| 14 | ICU capacity per hospital bed | Excluded | Similar to (13), though less independent of hospital bed occupancy |
|  | **Demographics** | | |
| 15 | Sex Ratio (M/F) | Excluded | Accounted for within SHMI |
| 16 | Mean Age | Excluded | Accounted for within SHMI |
|  | **Staffing** | | |
| 17 | Consultants | Excluded | Non-independent of hospital size |
| 18 | Consultant per bed | Excluded | Highly correlated with (21), performing a similar job role |
| 19 | Non-consultants | Excluded | Non-independent of hospital size |
| 20 | Non-consultants per bed | Excluded | As (19) |
| 21 | Total doctors per bed | Included | Sum of (19) & (21), studies associate doctor staffing and patient outcomes. A per bed metric makes the predictor relative to patient load. |
| 22 | Nurses | Excluded | Non-independent of hospital size |
| 23 | Nurses per bed | Included | Clinically relevant with evidence basis, perform unique role in patient care |

Appendix 2: Multivariate analyses assumption tests

To give validity to the results of a multiple regression analysis, certain assumptions should not be violated. The following assessment of the seven assessed assumptions show none appear significantly violated. However, of note within assumption 4, there appears one instance of non-linearity which should be accounted for on interpretation of the results.

Assumption #1: The dependent variable is continuous.

While this may be addressed, such as by transforming the data, or adapting the regression methodology (e.g. ordinal regression), it is not required in this circumstance.

Assumption #2: There is no multicollinearity in the data

We can test whether the predictors are too highly correlated in two ways. The first, an assessment of their Pearson correlations with any over or around 0.8 potentially being problematic (appendix 7). Comparing each year no predictor is correlated r= >0.8. The highest for each year is Nurses per bed vs. doctors per bed for 2019 and 2011 (r=0.721 & r=0.793, respectively), and ICU beds vs. doctors per bed in 2015 (r=0.572). The next highest in variously either doctors vs. nurses, ICU beds vs. doctors, or ICU beds vs. nurses (circa. r=0.55-65).

Secondly, can analyse a metric of collinearity called the Variance Inflation Factor (VIF). VIF over 10, with a tolerance over 2, are judged significant. If these thresholds are breached, an analysis of Eigenvalues can be undertaken to identify where the multicollinearity is arising from within the predictors. Neither in 2011, 2015, nor 2019, were these conditions met.

Thus, we can conclude that little or no multicollinearity exists in the data; though it is worth considering those predictors which are most correlated. This is especially true for doctors and nurses per bed, and the difference between the UVA and MVA for the latter. Given their correlation, it is difficult to disentangle how the variance in each of these predictors contribute to the observations.

Assumption #3: The relationship between independent and dependent variable is linear

Scatter plots of SHMI and the independent variables were undertaken, with a linear fit line added to each. A linear relationship was only not observed between the FHATB and SHMI in 2011, when trust performance on this metric was strongly homogenous. Log and square root transforms were attempted, but without success. Given the individuality of this broken assumption, a different methodology was not chosen (e.g. non-linear regression), but rather the author should interpret this result with caution. In further years, as performance became more heterogenous across trusts, a linear relationship was established between it and SHMI.

It is also worth noting that although a weak linear relationship exists, significant homogeneity in performance is also seen within the ICU beds per 10,000 admits scatterplot, meaning the discriminatory ability of this variable may be limited.

Assumption #4: The variance of the residuals is constant (homoscedasticity)

A homoscedastic dataset is the condition whereby the variance of the error terms in a regression model is constant. Thus, suggests a level of consistency within the dataset and the inputted predictor variables well-defines the change in the dependent variable. Heteroscedasticity is looked for in an XY scatterplot of predicted and model residuals, shown by some kind of pattern within the data. Figure 1 shows that this does not appear to be present within our data.


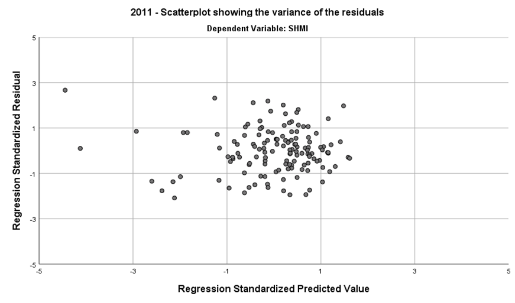

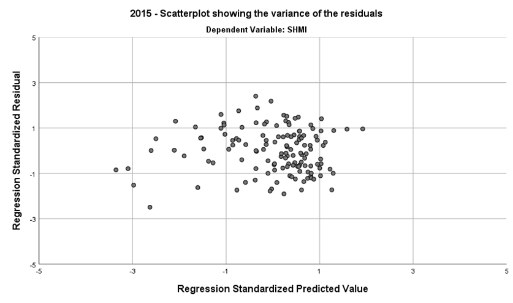

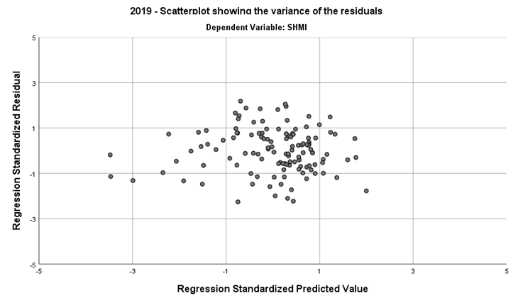


Appendix 2, Fig.1: A scatterplot of the predicted standardised residuals versus actual standardised residuals for all three analysed years, with SHMI as the dependent variable.

Assumption #5: The values of residuals are normally distributed

This can be done using both a histogram and a Normal P-P plot. Figure 2 demonstrates that each set of residuals appears approximately normally distributed, with minimal overall deviation from the normal P-P plot line.

Assumption #6: No influential cases bias the model

A

B


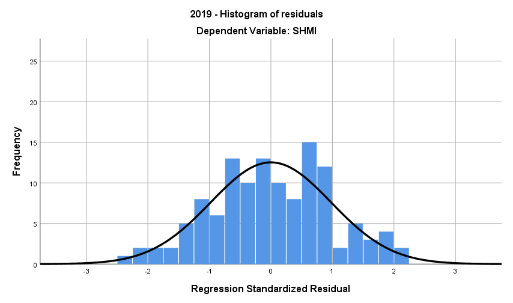

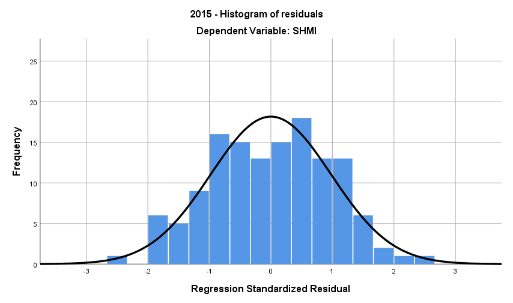

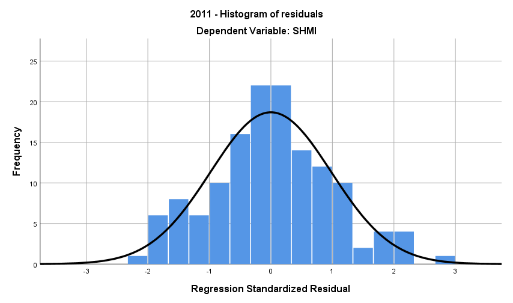

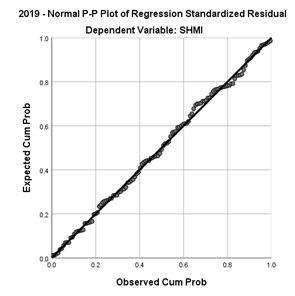

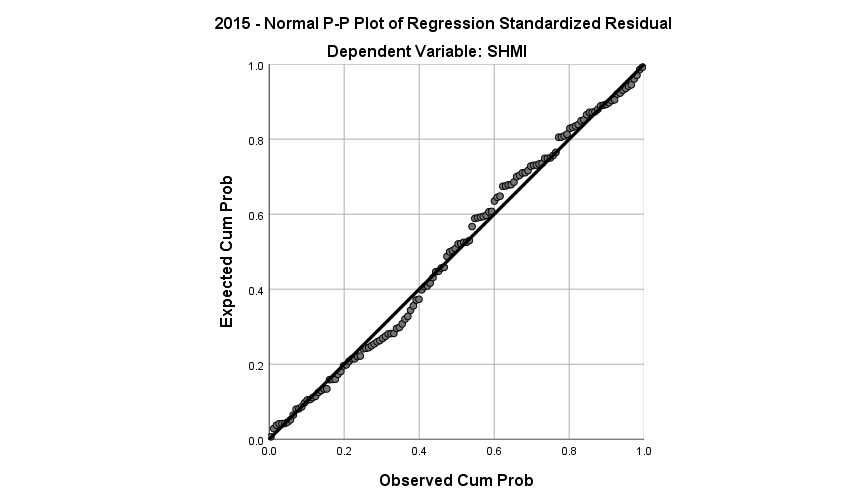

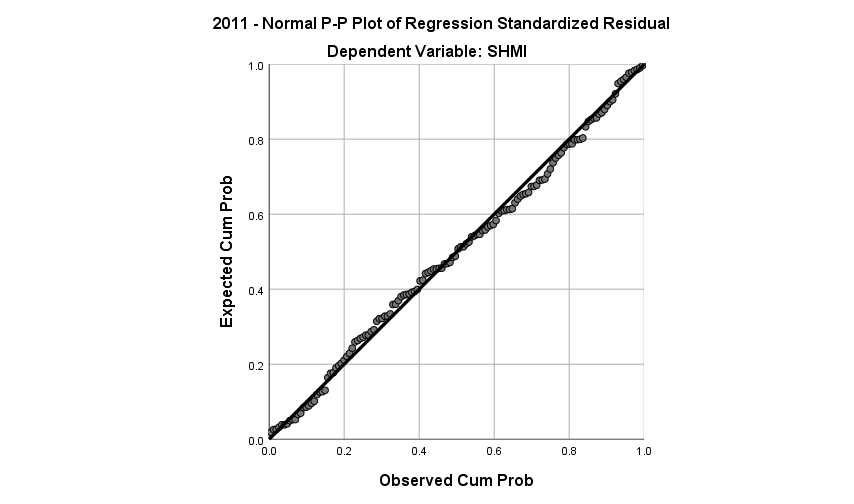


Appendix 2, Fig.2 A: Histograms of the standardised regression residuals B: Normal P-P plots of the standardised residuals

Observations within the dataset which have an undue influence on the regression should be accounted for. These can be identified by searching for significant outliers, high leverage points or highly influential points.

*Influential points*

Influential points can be assessed using Cook’s Distance. This is the difference between the predicted value from the regression with and without an individual observations. Thus, an influential observation has a large Cook’s Distance.

There are three approaches to the way in which Cook’s Distance can be used to identify influential residuals. The first is that any maximum Cook’s Distance under 1 is acceptable. The second, that any Cook’s distance of 4/n (n being the number of residuals in the regression) should be investigated. Finally, that a visual analysis of the Cook’s relative to each other should be undertaken, so as to see whether one is substantially deviating from the others.

Table 2 shows that the first approach is satisfied for each year.

Appendix 2, Table 2: Values for the Cook’s Distance and Centered Leverage Values for the 5P multivariate analysis

For the second approach, Cook’s over the cutoff shown within table 9 identifies 13, 6 and 6 entries for 2011, 2015 and 2019, respectively.

Finally, using an XY scatterplot, obvious outliers can be identified in each dataset. These are shown in figure 3. The most striking outlier is in 2011 (RJZ), with the frequency and extremity of outliers appearing to reduce over time. There is some overlap noted in the outlying trusts, albeit with the consideration that the name, number, and configuration of trusts within the set have changed over time.


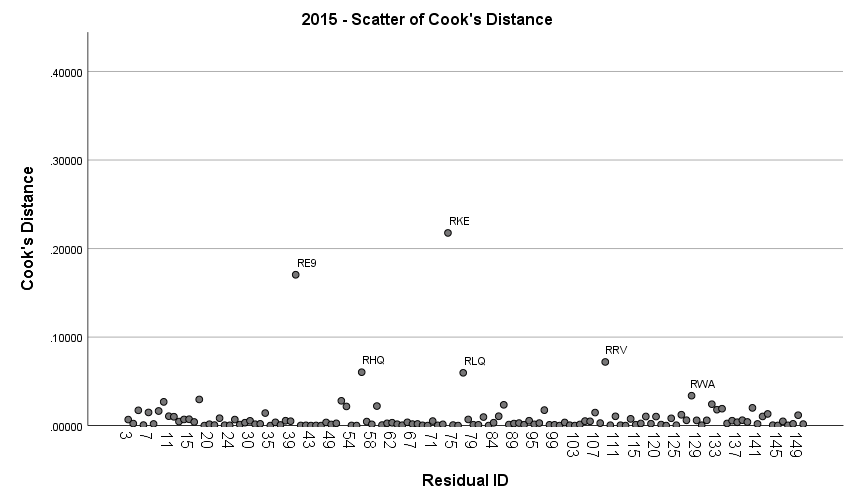

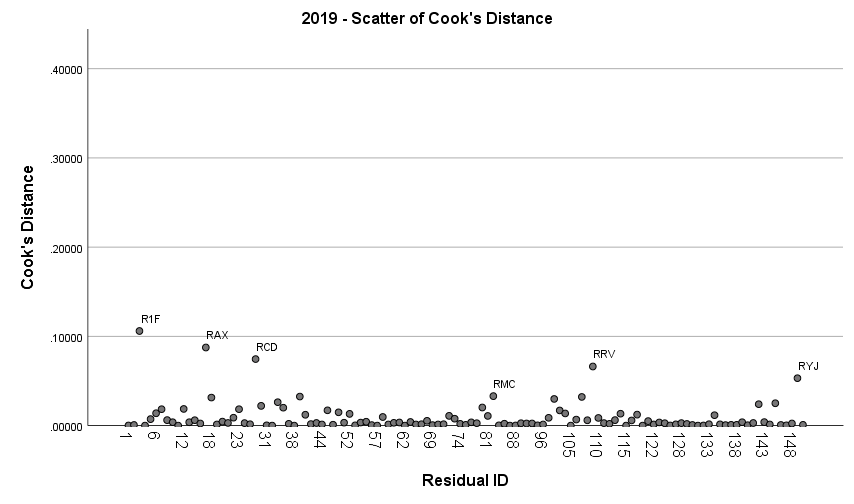

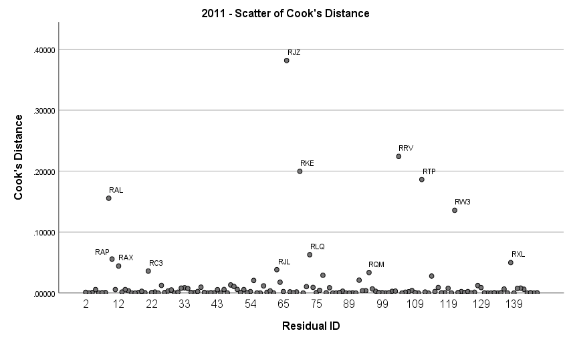


Appendix 2, Fig.3: Scatterplots of the Cook’s Distances by residual ID, for each year of analysis. The organisation code for outlying trusts are identified for those identified as outliers.

*High leverage points*

Leveraged values will have an abnormal effect on the regression line, by pulling the line towards it. The average leverage score is calculated as (k+1)/n, where k is the number of independent variables in the model. Observations with high leverage will have a score 3 times this value. As above, along with the numerical approach, a visual inspection can also be undertaken (figure 4). The former method identifies 6, 4 and 3 cases for 2011, 2015 and 2019, respectively.


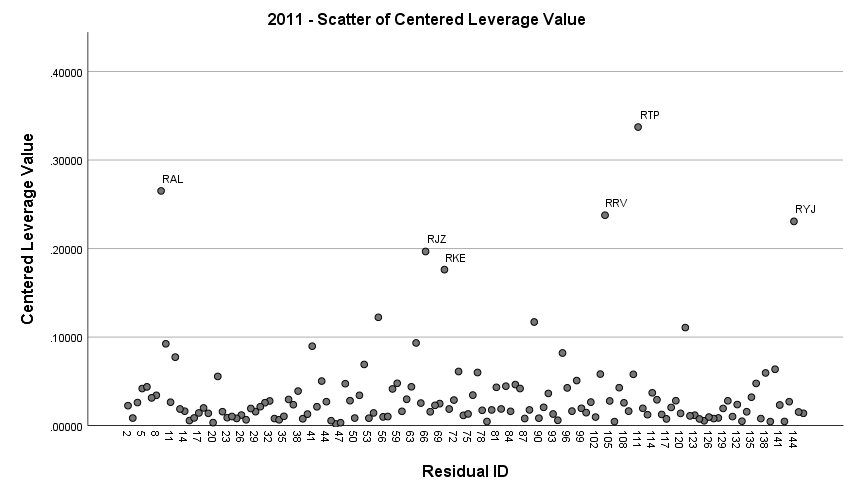

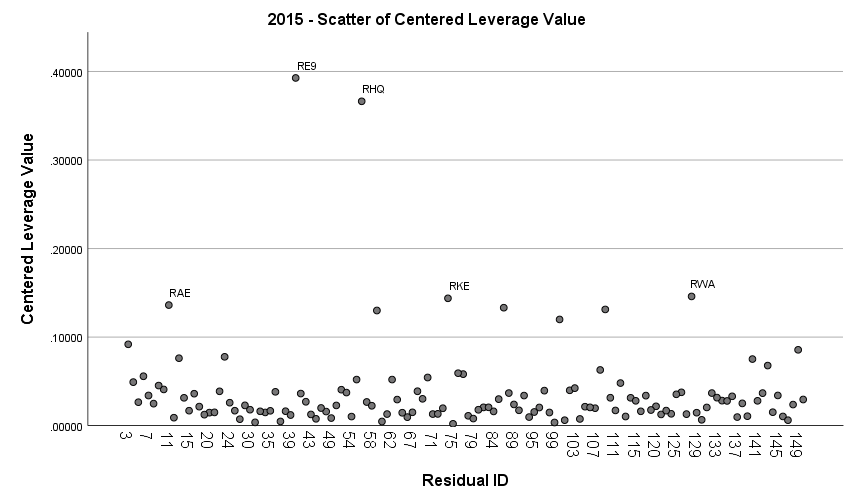

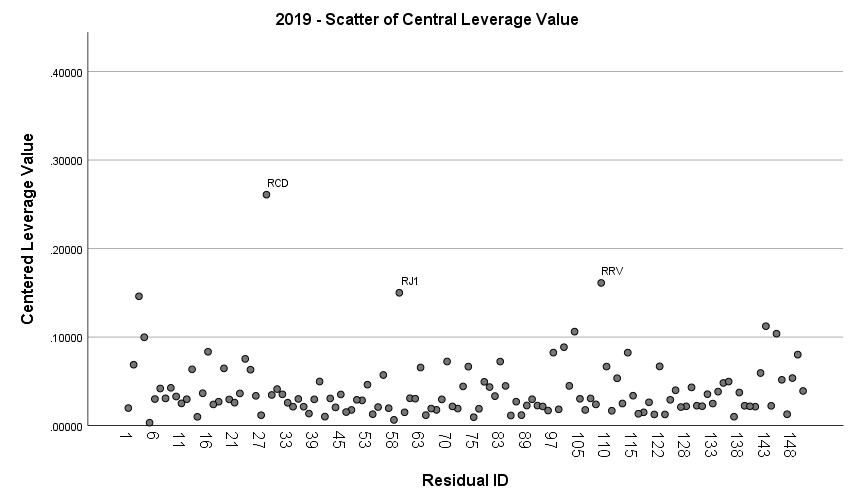


Appendix 2, Fig.4: Scatterplots of the Centered Leverage Values by residual ID, for each year of analysis. The organisation code for outlying trusts are identified for those identified as outliers.

*Combining Leverage and Cook’s*

The most worrisome cases can be identified by plotting the results of the Leveraged values against the Cook’s Distances (figure 5), showing those which both add variability to the regression estimates (Cook’s), and also affect the slope of the regression equation (Leverage).


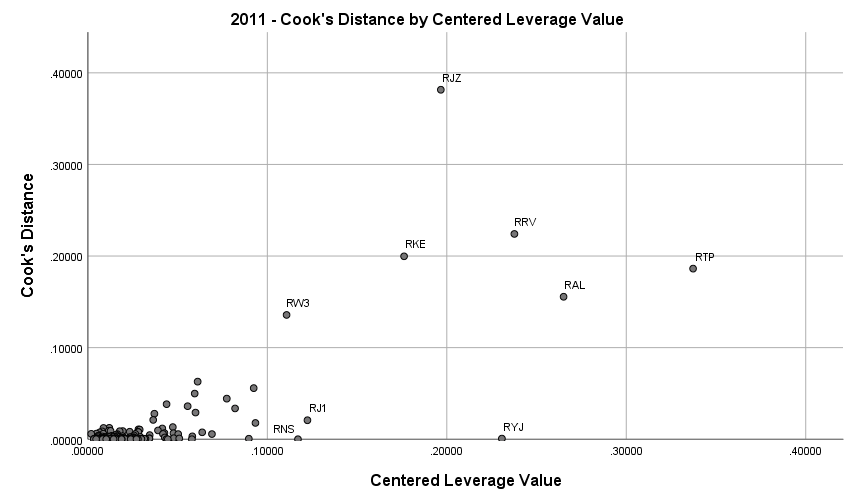

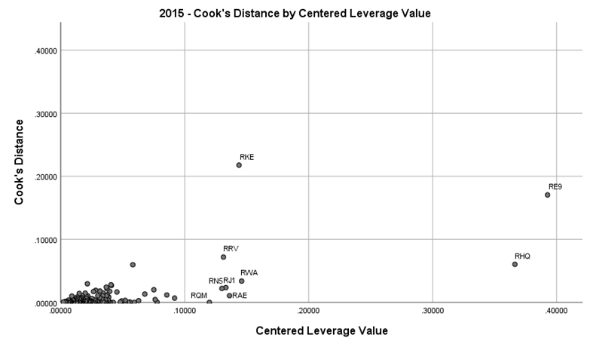

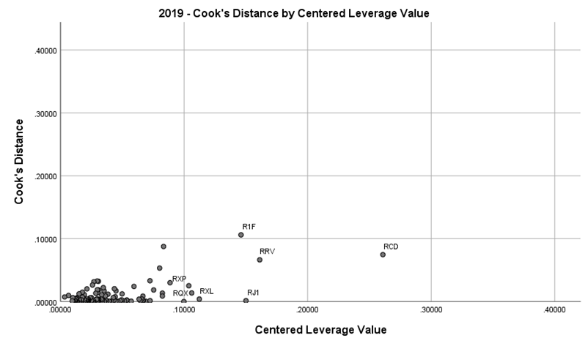


Appendix 2, Fig.5: Scatterplots of the Centered Leverage Values by Cook’s Distance, for each year of analysis. Here, the integration of both methods identifies those most concerning. The organisation code for outlying trusts are identified for those identified as outliers.

*Outliers*

Extreme outlier residuals will be of greater than ±3, meaning its observed frequency is significantly greater or less than its expected frequency and should thus be identified^152^. Table 3 shows how the standardised residuals for each year show that this threshold is not beached.

Appendix 2, Table 3: The statistics for the standardised residuals. The value of those residuals which have the greatest (maximum) and least (minimum) observed frequency compared to what is expected are identified. n: the number of residuals analysed.

It is also possible to identify potential outliers using the Mahalanobis distance. This indicates how far the case is from the centroid of all cases for the predictor variables. The significance of each distance is calculated, with any probability under 0.001 being significant and considered needing investigation of being a multivariate outlier. In graphical form (figure 6), this equates to any distance over 20. This identifies 6, 2 and 1 outlier for 2011, 2015, and 2019, respectively.


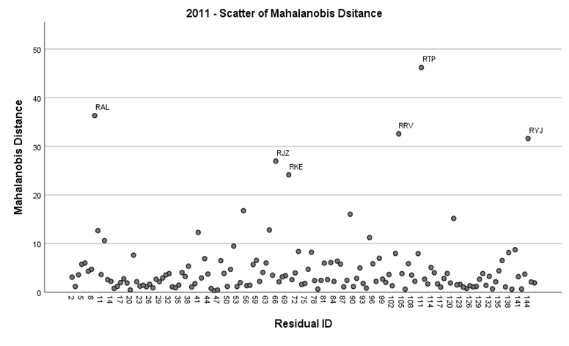

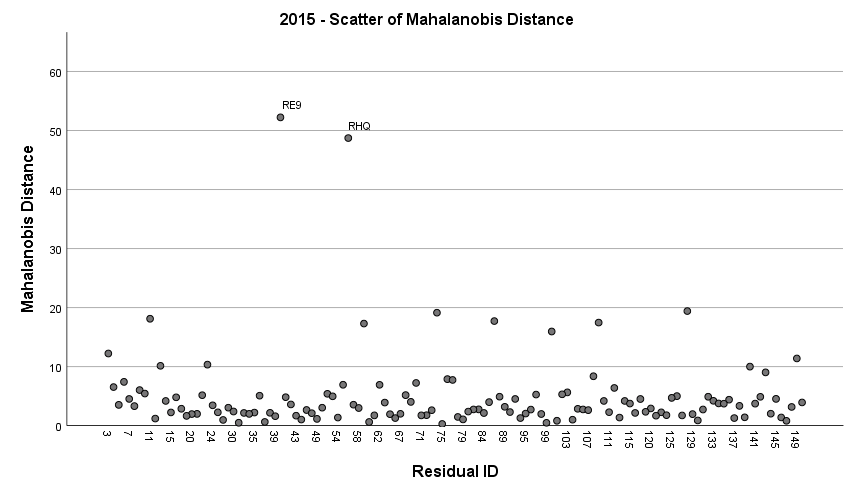

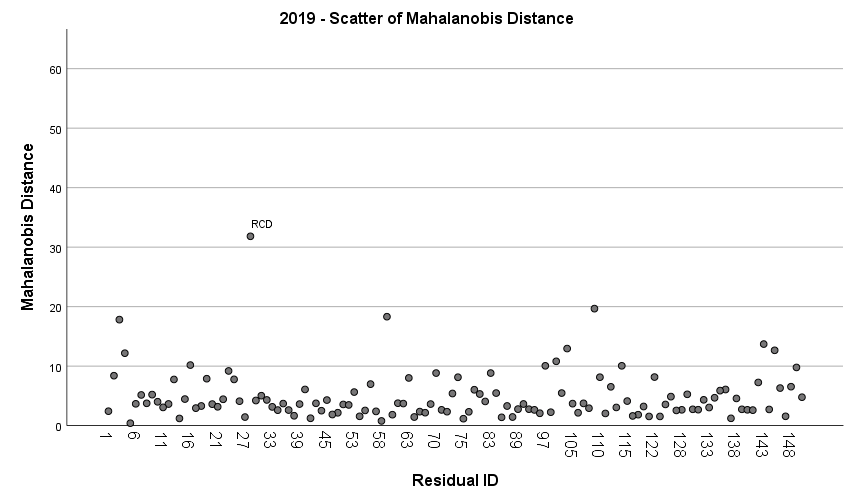


Appendix 2, Fig.6: Scatterplots of the Mahalanobis Distance by Residual ID, for each year of analysis. The organisation code for outlying trusts are identified for those identified as outliers.

*Consensus*

The concerning entries were assessed, and a consensus decided on the basis of being flagged by more than one methodology, on the basis of both statistical test and visual analysis (table 4).

Appendix 2, Table 4: A summary table of the trusts highlighted as potential outliers by the methods described. ‘Clear’ outliers were those identified by the statistical methodology, along with a visual assessment of the scatterplots, while those ‘marginal’ outliers just the former

Appendix 3: A description of the robustness testing undertaken

*Outliers*

In order to ensure that the entries of concern were not significantly effecting the interpretation of the results, the MVA were repeated with the highlighted entries above excluded (appendix 5). For each year, the adjusted R² value is somewhat reduced. Those predictors which were statistically significant remain the same, and equally the standardised coefficients are similar. Therefore, it appears that the presence of these concerning outlying entries did not have an egregious effect on the results of the MVA.

*Common data*

It is possible that the changing composition of hospital trusts over time could have affected the observations of the analysis. To exclude this, the MVA was repeated on only the 117 common to all three years (appendix 6). Reassuringly these results are consistent with the ‘5P’ MVA, with coefficients of a similar size and sign. Notable differences include that the FHATB has a greater coefficient size for all three years and is now also statistically significant for 2015. In contrast, while the coefficients are of similar size, bed occupancy is now not statistically significant in 2015, although the absolute change in *p*-value is modest. Finally, nurses per bed has a notable increase in coefficient in 2019.

The R² value increases in the common set MVA for all three years, and particularly so for 2011 (average ‘5P’ 0.364 vs. common set 0.393). This could be accounted for by an increase in the effect size of doctors per bed in 2011. While small, this predictor has the greatest size and thus the marginal effect may be exaggerated compared to others.

These data would suggest that the associations observe within the 5P MVA were not affected by the changing composition of trusts included within each analysis. Indeed, some element of the associations may have been masked.

Appendix 4: Normality assessments

A visual representation of the normality of each variables’ distribution, combining values for each Trust included in the analysis and displaying them in a histogram fitted with a bell curve. The respective axes and binning have been standardised for each variable over the three years as possible.


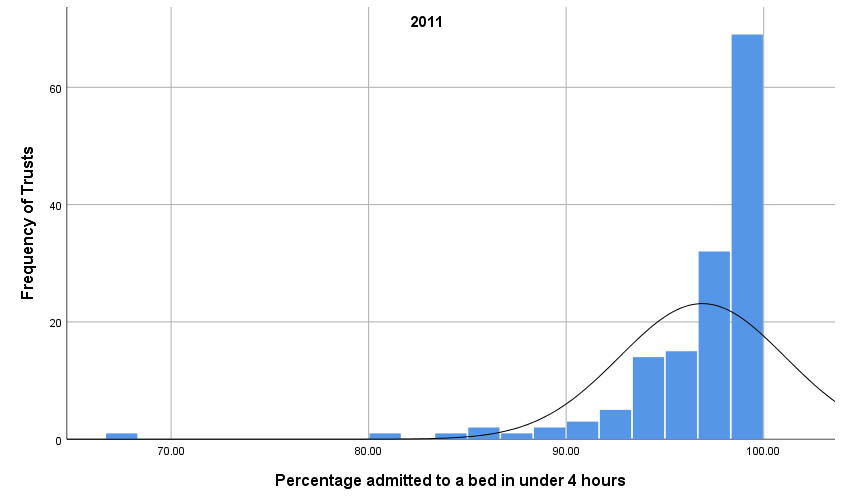

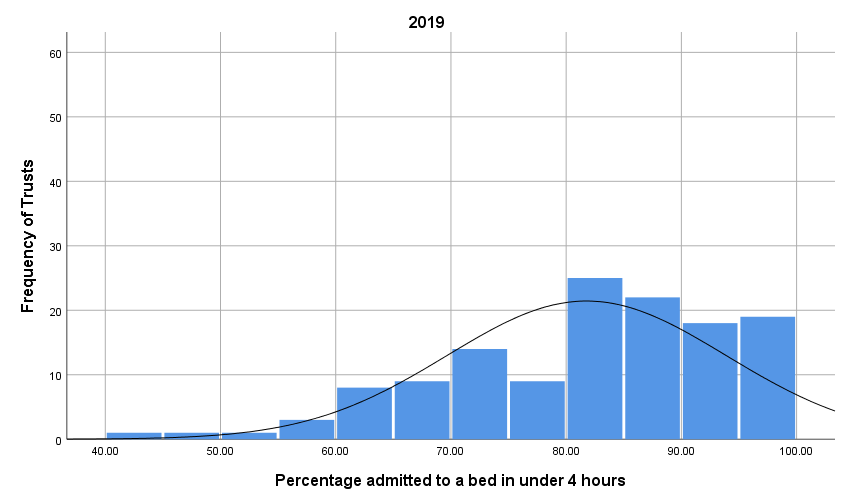

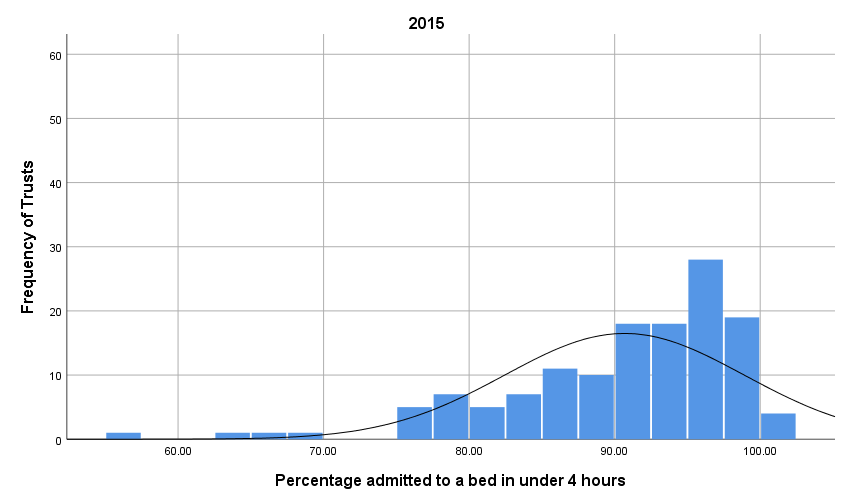

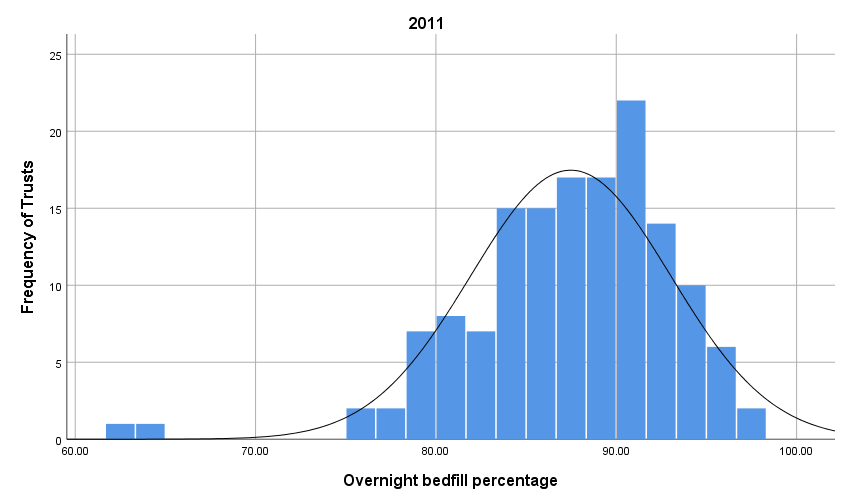

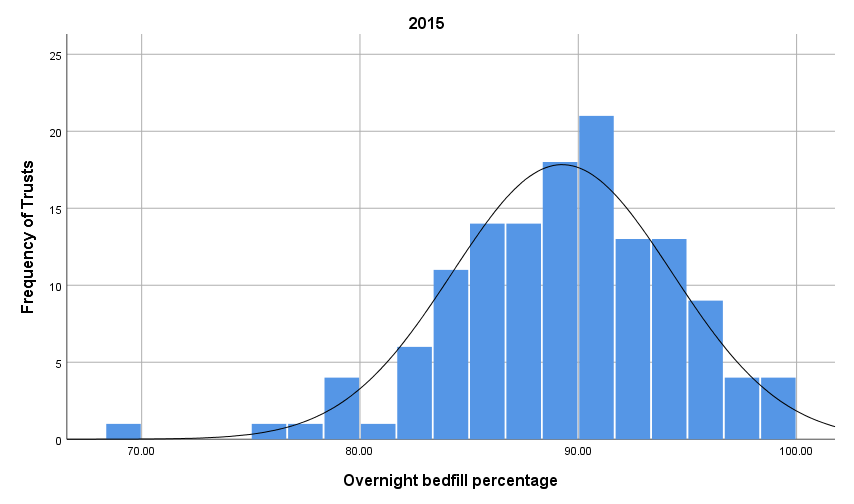

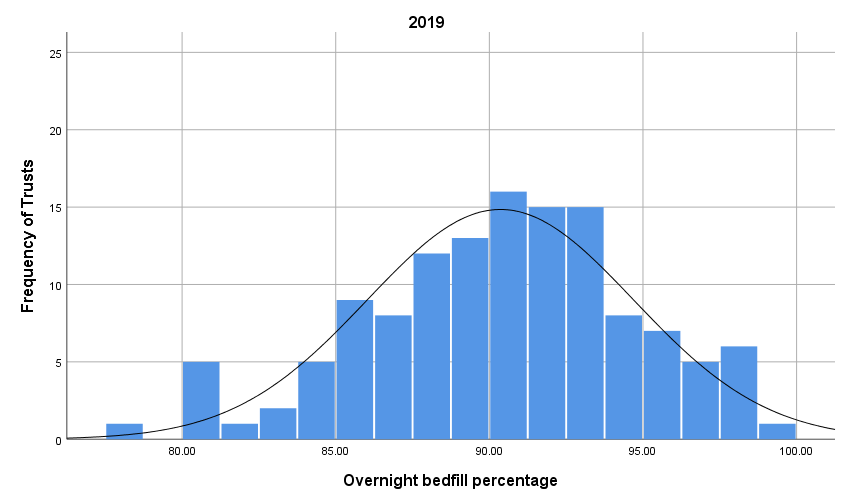

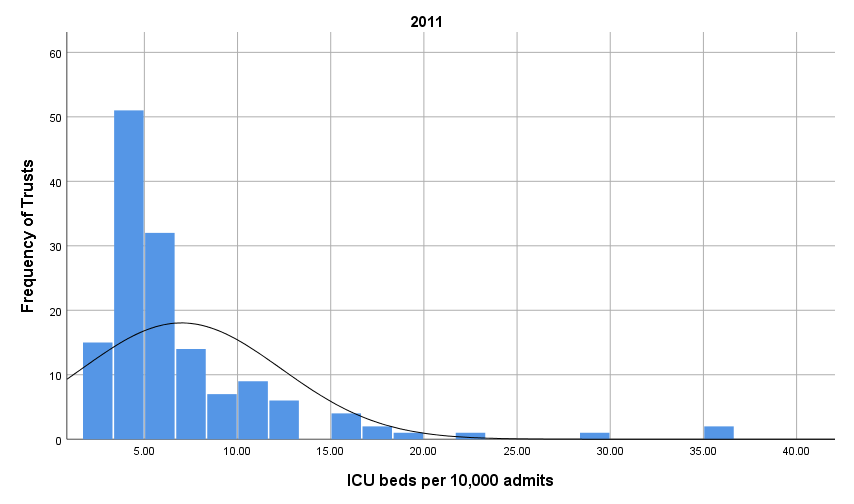

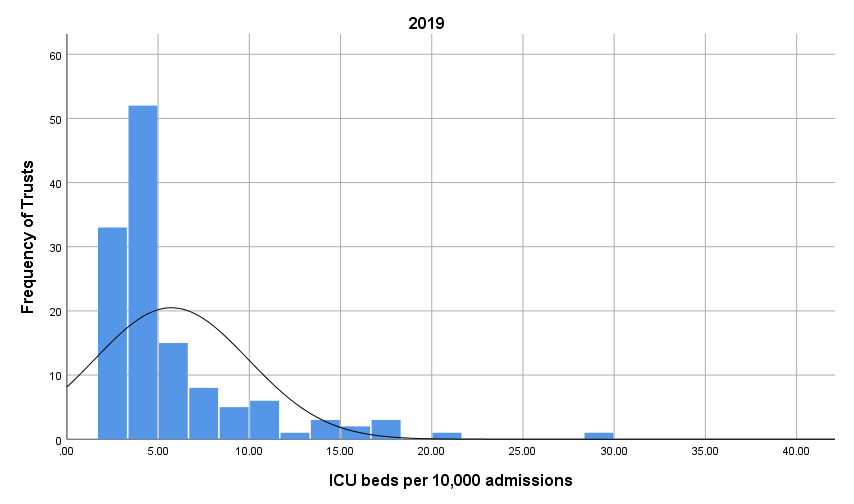

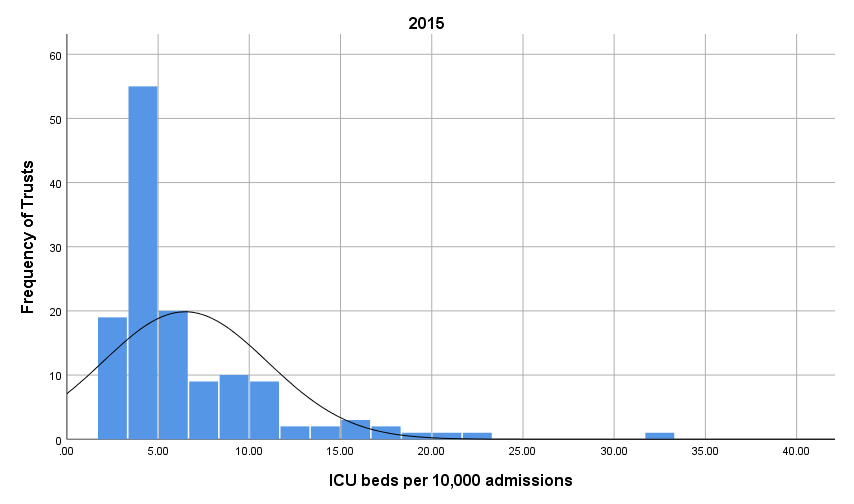

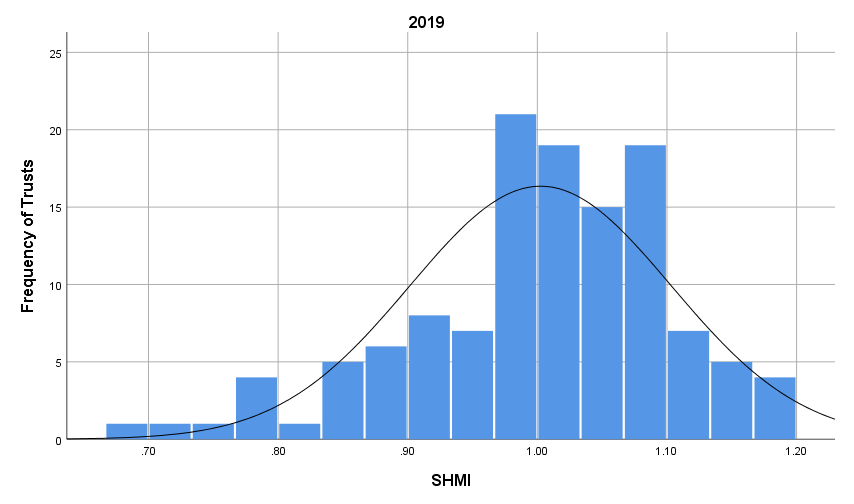

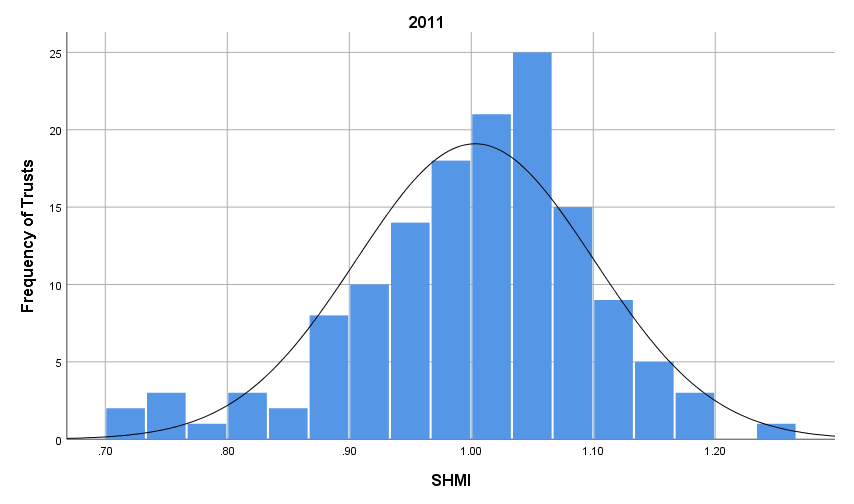

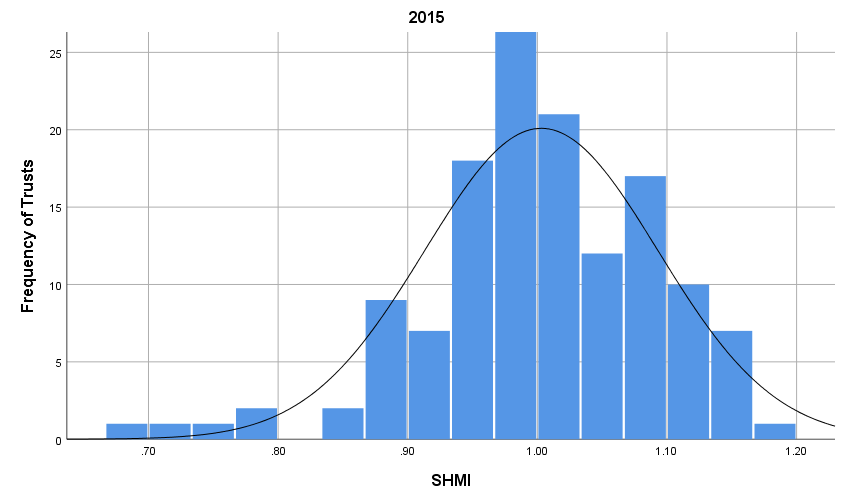

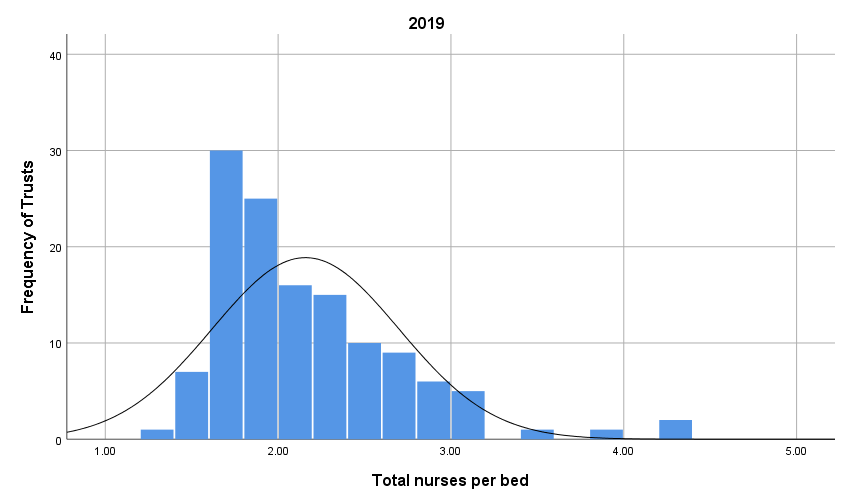

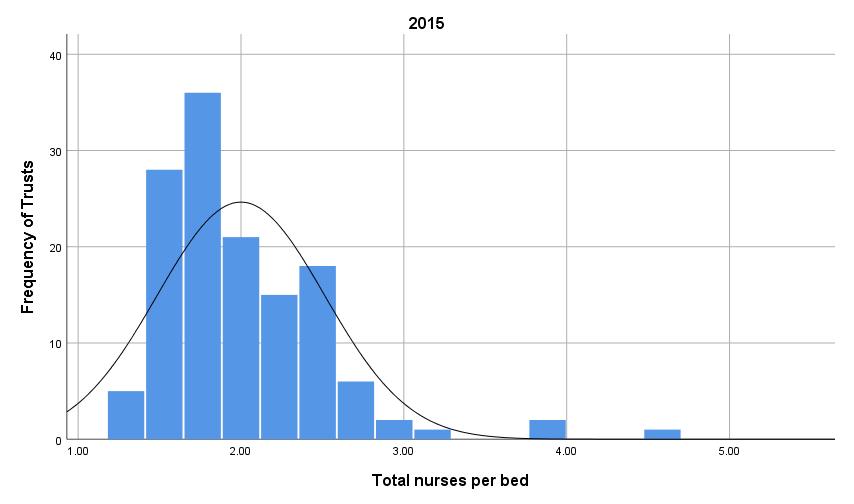

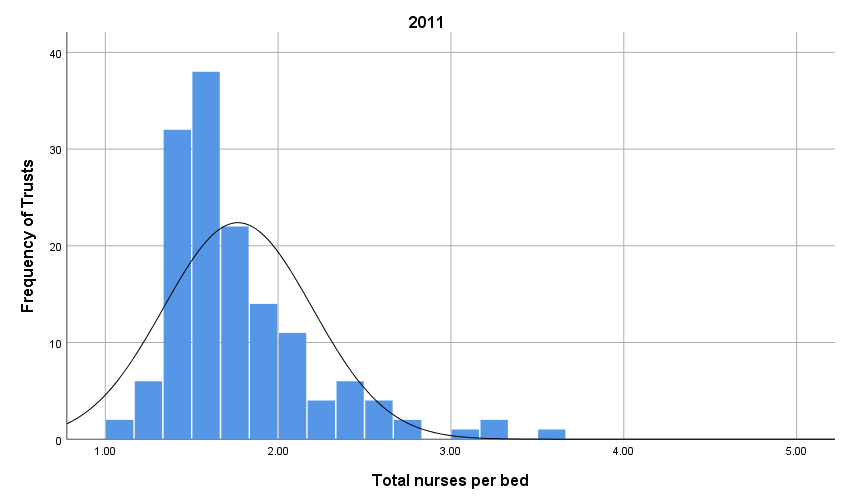

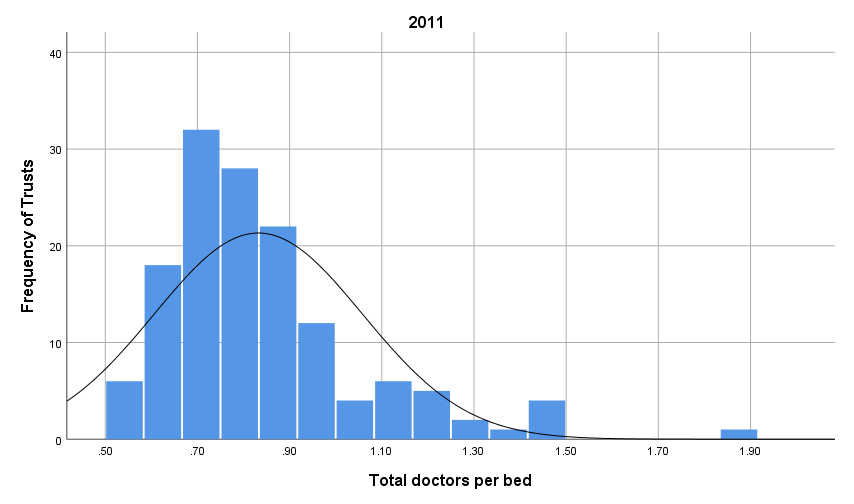

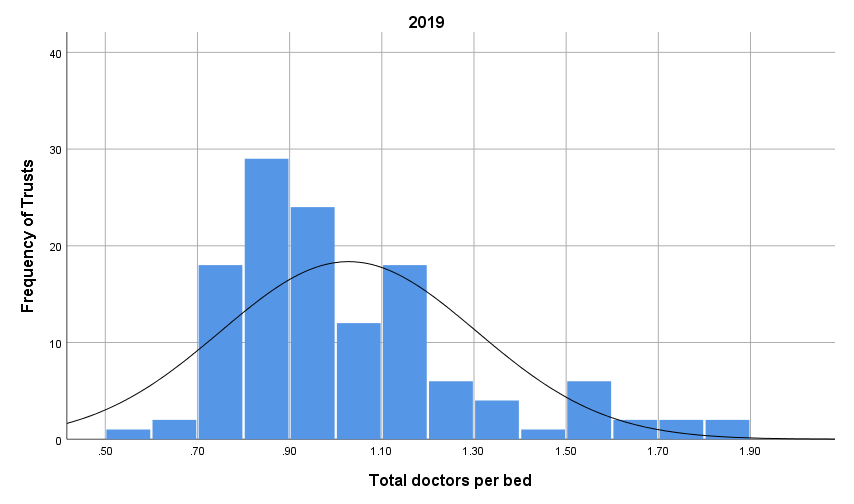

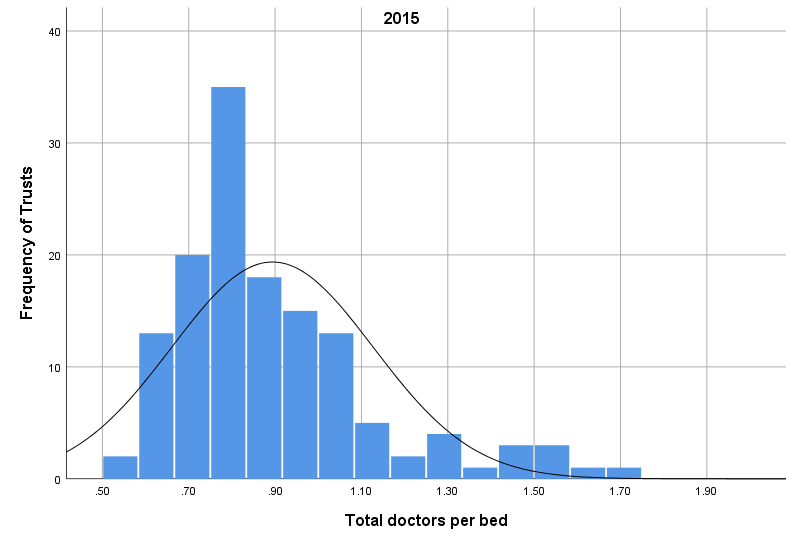

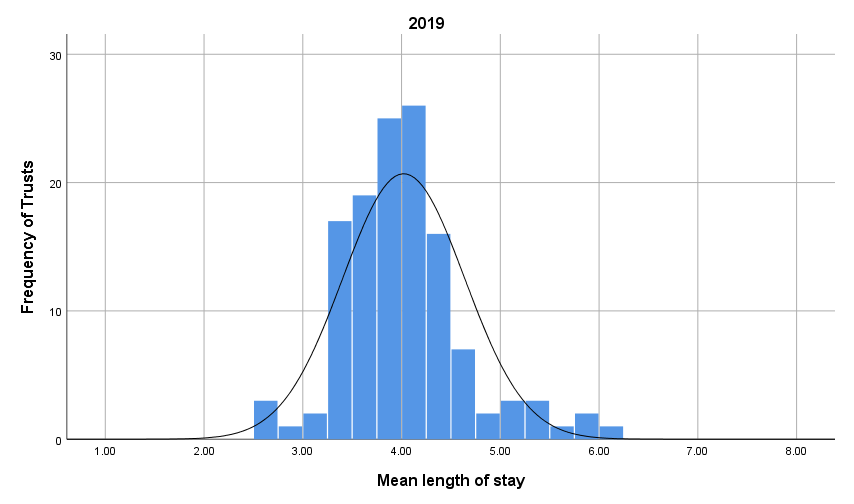

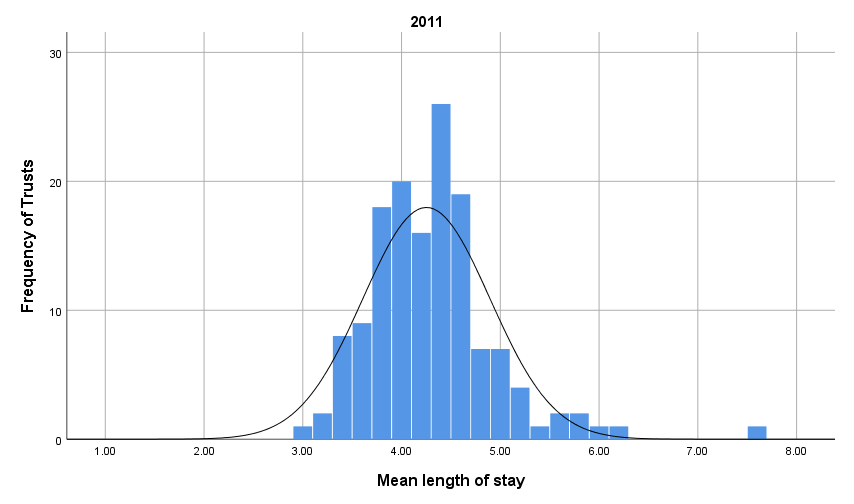

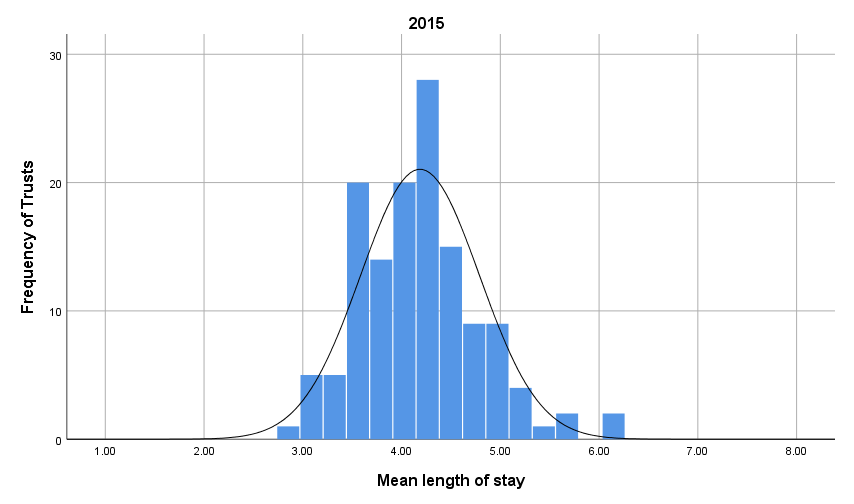

Appendix 5

Appendix 5: The results of the 6-independent predictor ‘6P’ multivariate regression analysis, comparing their association with SHMI. Significance judged as *p*=<0.05. Unstd.: unstandardised, Stnd.: standardised, Sig.: significance

Appendices 6 & 7: MVA results from robustness testing

Appendix 5: A repeat of the 5-predictor (‘5P’) multivariate analysis with the identified outlying trusts excluded from the analysis. Significance judged as *p*=≥ 0.05. Unstd.: unstandardised, Stnd.: standardised, Sig.: significance

Appendix 6: A repeat of the 5-predictor (‘5P’) multivariate analysis using only those trusts common to all three years of each cross-sectional analysis. Significance judged as *p*=≥ 0.05. Unstd.: unstandardised, Stnd.: standardised, Sig.: significance

Appendix 8: A correlation matrix of included predictors
